# Breastfeeding experiences and women's self-concept: Negotiations and dilemmas in the transition to motherhood

**DOI:** 10.3389/fsoc.2023.1130808

**Published:** 2023-04-05

**Authors:** Amélia Augusto, Dulce Morgado Neves, Vera Henriques

**Affiliations:** ^1^ISCTE - Instituto Universitário de Lisboa (ISCTE-IUL), Centro de Investigação e Estudos de Sociologia, Lisboa, Portugal; ^2^Departamento de Sociologia, Universidade da Beira Interior, Covilhã, Portugal

**Keywords:** breastfeeding, transitions to motherhood, feminism, identity work, Portuguese mothers, focus group

## Abstract

**Introduction:**

Breastfeeding is much more than a biological event. It is a social construction, full of cultural meanings and framed by social structures. Being, simultaneously, a natural event and a social practice, breastfeeding poses challenges to feminist approaches in the sense it may be acknowledged as an empowering practice for women and/or as a setback in the process of women's social emancipation. Often focused on the product, i.e., the milk and its beneficial properties for the infant's health, the dominant discourse on breastfeeding makes it a trait of good mothering, withdrawing the understanding of the particular (but also structural) contexts in which this practice occurs.

**Methods:**

Based on results from a focus group with five mothers of a first child, this paper addresses first-person testimonies about breastfeeding and transition to motherhood, aiming to capture eventual self-concept dilemmas, impacts of social judgments, difficulties related to the work-family balance, as well as negotiation processes taking place within couples and early-parents.

**Results and discussion:**

Despite being subject to tensions and sometimes stressful adaptation processes, motherhood and breastfeeding tend to be ultimately described by women as experiences that enhance welcome changes in personal trajectories, life priorities and identities.

## 1. Introduction

### 1.1. Literature and research on breastfeeding: Current debates

The whole process associated with motherhood—pregnancy, preparation for childbirth, childbirth itself, and breastfeeding—has been the subject of strong social valorization and growing social expectations. When they become mothers, women experience a set of pressures to succeed at each stage of that process. Regarding breastfeeding, there is a predisposition to understand it as a natural extension of childbirth, constituting a mandatory stage in the process of raising a child. Often considered as a biological and natural event, breastfeeding is in fact a social practice that cannot be understood outside the historical time, the social class, and the political and cultural context in which it occurs. Understanding the objective and subjective experiences of breastfeeding, the production of social and individual meanings which it entails, the dilemmas and demands faced by women who breastfeed, therefore, requires an understanding of the social determinants that shape this reality. It also requires analysis of the broader social contexts within which particular perceptions and meanings are constructed, and the interests and powers that operate in the definition of what is socially expected and desirable in this regard. Breastfeeding is a socially constructed and culturally variable phenomenon, requiring a sociological and critical deconstruction of the naturalized, normalized, and neutral character that is commonly attributed to it.

In general, the most diverse discourses endorse a strong social valorization of breastfeeding, understood as beneficial for the babies' health, for the wellbeing of babies and mothers, as well as for the reinforcement of the bond between both—an approach that, to great extent, finds its anchorage in medicine. The World Health Organization (WHO) states that breastfeeding in developed countries should be exclusive for 6 months and continue, along with solid foods introduction, up to 2 years or beyond (World Health Organization, 2003). As a result of such recommendations, “in the nineteen nineties breastfeeding started to be considered the norm, a matter of public health that should be preserved for the benefit of future generations” (García et al., 2019; p. 232).

Given a strong pro-breastfeeding discourse, which clearly goes under the banner of freedom of choice (García et al., 2019), there is a socially widespread idea that the ability to exercise choice to breastfeed is as simple as deciding to do so (Andrews and Knaak, 2013). However, breastfeeding does not exist separately from the other dimensions of mothers' lives, and, therefore, social and structural factors that act as barriers to breastfeeding must be taken into account, considering that not all mothers are on equal footing to exercise their “choice,” particularly for exclusive (up to 6 months) or prolonged (up to 2 years or more) breastfeeding, as recommended by the WHO. Often, feminist approaches call attention to the problematic nature of configuring breastfeeding choices as if they are individual and autonomous. Poststructuralist feminist perspective highlighted some contradictory social constructions and practices faced by women when negotiating infant feeding in contemporary Western contexts (Johnson et al., 2009).

Van Esterik (1994) stated that, although breastfeeding is recognized as a woman issue, it is seldom framed as a feminist issue, as it is often ignored by feminist theories. According to this author, that happens because breastfeeding raises conceptual problems and reveals many of the contradictions that feminism struggles with. However, rather than the absence of breastfeeding in feminist theories, we may speak of a diversity in the feminist analyses, which arises, in part, from an ambivalence about the extent to which breastfeeding should be considered a source of power (and therefore of celebration) or oppression for women.

McCarter-Spaulding (2008) considers that when breastfeeding is scrutinized with a feminist lens, it becomes somewhat problematic. The problem, she considers, arises because breastfeeding is sex specific, and therefore challenges the feminist principle of gender-neutral child-rearing. Given the social roles associated with motherhood, breastfeeding can be potentially oppressive to women. From a liberal perspective, breastfeeding can be seen as a gender difference that accounts for a further burden to women, standing in the way of their liberation. Law (2000) endorses this perspective, considering that breastfeeding can contribute to the gendering of domestic labor, by focusing on the baby, excluding women from the labor market, and getting women back to the domestic and private space. In principle, few feminists will be against motherhood and breastfeeding, their concerns here are the dominant gender regimes regarding family duties in the context of a patriarchal society.

Other feminist standpoints tend to see women's bodies and their reproductive functions as sources of spirituality and power, rather than as sites of oppression. Women's innate biological monopoly over certain aspects of the reproductive process is actually celebrated by some feminists as a particular power that needs to be defended and preserved (Jackson, 2001). Contemporary radical feminism abounds with references to the “inherent power of female biology” and the “creative power” that is associated with female biology, as well as the “innate talent and superiority of women” (Shilling, 1993; p. 60). This sort of statement implies the suggestion that women's special power lies in their closeness to nature, provided by their ability to give birth. This naturalistic insight into the body reflects the exaltation and celebration of the virtues granted to women by nature.

The romanticization of motherhood and breastfeeding fails to take into account the social, cultural and material conditions under which women become mothers and breastfeed. By celebrating their biological functions and natural attributes, discourses contribute to the essentialization of women (Van Esterik, 1994). Schmied and Lupton (2001) criticize the romanticizing of maternal identity, based on the idea of an “authentic” or “true” feminine self that can be experienced and discovered through breastfeeding. According to them, the feminist framework that promotes breastfeeding as a source of female empowerment and alternate subjectivity limits the acknowledgment of the difference and diversity among women. Difference and diversity, we stress, that are permanently highlighted by the embodied experiences of women, although their apparently common biology: “breastfeeding forces us to think about women's bodies, and thus the other aspects of their bodies, race, age, health status, class, position, sexuality that define women's experience and circumscribe their mothering practices in the context of male-dominated societies” (Hausman, 2004, p. 275).

The promotion of breast milk as “nutritionally superior” endorsed by health movements became progressively more medicalized as it developed into a focus on the specific nutritional properties and health outcomes of breast milk (Torres, 2014a). Women and their bodies have been a particular target of medicalization, partly due to gender-based ideologies that see women essentially as reproductive beings, which has led to the medicalization of the female reproductive process (Ragoné and Willis, 2000). The clinical gaze has progressively broadened to include menstruation, pregnancy, childbirth, breastfeeding and menopause. As part of this widening, breastfeeding came to be constructed as a medical issue, contributing to further control over motherhood.

This medicalized construction of breastfeeding places the focus on the product (breast milk) and not on the process. By emphasizing the nutritional properties of breast milk and its outcomes for the baby's health, it does not consider the social and emotional aspects of breastfeeding (Torres, 2014a). Law (2000) refers to tensions on conceptualizing breastfeeding as biological reproduction (lactation) vs. social reproduction (breastfeeding). The author argues that breastfeeding is about social, domestic, and technical arrangements, rather the nutritive qualities of breast milk as opposed to formula. According to his perspective, lactation is a biological phenomenon that takes place in the human body, but breastfeeding is a form of social labor that must be negotiated.

Andrews and Knaak (2013) refer to authors who have drawn attention to the medicalization of infant feeding, pointing out how breastfeeding, while considered a natural event, must nevertheless be taught to mothers through scientifically based, professional intervention. This discourse of expertise, which implies the need for close expert supervision, places new restrictions on mothers' choices about how to breastfeed and for how long (Carter, 1995; Blum, 1999), further contributing to feelings of guilt by mothers who do not conform to the norm, either because they cannot or don't want to. According to Jackson et al. (2021), formula feeders experience external guilt most commonly from healthcare professionals, whereas breastfeeding mothers experience guilt most commonly associated with peers and family. Actually, emotions women often feel regarding infant feeding may be better conceptualized as shame, since it can involve a negative self-assessment resulting from the failure to achieve an idealized notion of good motherhood (Taylor and Wallace, 2012; Thomson et al., 2015; Jackson et al., 2021; Doonan, 2022; Naylor, 2022; among others). Also Badinter (2010) discusses the emergence of a new orthodoxy—the “Good Motherhood”—that emphasizes natural practices, such as long-term breastfeeding, washable nappies, organic food, which compels women to allocate large periods of time and much of their energy to childcare. For the author, this subscription to motherhood, which resides in the validation of nature as opposed to culture, does not help the feminist cause; quite the contrary (Faircloth, 2017).

Medical discourses, although stressing the bonding between the baby and the mother, predominantly frame this connection around biological or natural account of symbiosis, underlining the anatomic functioning of the breast and the production of breast milk (Schmied and Lupton, 2001), subscribing a disembodied version of women. This focus on the “natural” and biological aspects of breastfeeding leaves no room for the acknowledgment of its multidimensionality: “breastfeeding is a holistic act and is intimately connected to all domains of life—sexuality, eating, emotion, appearance, sleeping, parental relationships” (Van Esterik, 1994, p. S42).

It is important to explore and understand the lived experience of breastfeeding not as the result of an individual act or choice, but by reference to the different components of women's lives, taking into account gender inequalities, gender roles related to care, the return to work after birth, the social expectations they feel they have to meet, the time and energy that the process demands, the pain and discomfort it may causes. Basically, “if breastfeeding is political, it is so in relation to a feminist politics that can focus on what makes mothers particular kinds of embodied citizens, with needs, rights, and perspectives of public good” (Hausman, 2004, p. 275). We need approaches that allow us to understand women's lived experiences of breastfeeding and to explore the discourses on which they rely when articulating and making sense of these experiences (Schmied and Lupton, 2001).

### 1.2. An overview on the Portuguese context

In Portugal, the participation of women in the labor market is a distinctive feature of the national socioeconomic character. In this country, the female employment rate is relatively high, and it is particularly contrasting when compared to other Southern European countries. According to official data for 2021 (Labor Force Survey, INE), Portugal has 2.4 million working women, the equivalent to half of the employed population. Most employed women are mothers and have completed higher education.

Female employment is continuous, as most women do not interrupt their professional activity after becoming mothers. Also, Portugal stands out from other EU countries with the prevalence of a dual-earner model fundamentally based on full-time employment (Marques et al., 2021): while, in Portugal, female part-time employment did not go beyond 9.8% in 2021, for EU27, the figure was about 30%. This expressive participation of women in the labor force cannot be dissociated, on the one hand, from the persistence of an economy based on low wages (which makes particularly difficult to families to economically sustain the households with just one salary), and, on the other hand, from the importance that Portuguese women attribute to their economic independence.

In Portugal, public policies regarding parental leave have undergone important changes in recent decades. As in other European countries, these policies have not only reinforced the duration and compensation of leaves but have also introduced new provisions to promote greater gender equality in the family-work conciliation, children wellbeing, and birth rates (Wall et al., 2019). Currently, parental leave assumes a maximum duration of 120 consecutive days (4 months) paid at 100%, with financial penalties from that period onwards.

Concerning breastfeeding supporting policies, the current Portuguese Labor Code enshrines the right of a daily leave, up to 2 h, for breastfeeding during the child's first year of age or for as long as breastfeeding lasts, through medical certificate from 12 months onward.

In line with international recommendations, the Directorate-General for Health and the Portuguese Pediatric Society recommend exclusive breastfeeding until the age of 6 months. However, the prevalence of breastfeeding in Portugal remains lower than intended and with an important decrease in the first months of life. There are not many available statistics on the prevalence of breastfeeding in the country, but some studies (Sandes et al., 2007; Brito et al., 2011) estimate that more than 90% of Portuguese mothers initiate breastfeeding after birth. However, almost half of these mothers give up breastfeeding during the baby's first 3 months.

The return to work is one of the factors, pointed out by mothers, that most contributes to the early cessation of breastfeeding and to the non-compliance with their breastfeeding projects. The reduction in pay after 4 months of parental leave is certainly a barrier, for many families, in complying with the WHO recommendations regarding exclusive breastfeeding until the baby is 6 months old.

## 2. Methods and materials

### 2.1. Focus group

This exploratory research is based on data resulting from a focus group with Portuguese mothers who declared to have breastfeed (or being breastfeeding) their children. Focus group is a collective discussion technique, where a group of selected individuals share opinions and experiences related to a particular subject (Wilkinson, 2004). To some extent, focus group discussions intend to resemble natural social interaction, as this technique stimulates the sharing of meanings related to an experience that is common to all participants. The goal is to create a familiar, non-threatening and inclusive environment, where participants feel comfortable to discuss their opinions and experiences.

In this case, focus group took place in an online platform (Zoom), and it lasted ~2 h and 15 min. The collective interview was conducted by one of the first authors, coadjuvated by the other, and it followed an open script, aiming to capture challenges imposed by breastfeeding and other embodied practices of childcare, exploring self-concept dilemmas, impacts of social expectations and judgements, difficulties related to the work-family balance, as well as negotiation processes taking place within couples and early-parents.

### 2.2. Participants

A call for participation in the focus group was launched in social media (Facebook and Instagram), introducing the main objectives of the study as well as the criteria for the selection of participants. Three criteria were previously defined: to live in Portugal; to be mother of a first child up to 3 years old; to have breastfed/be breastfeeding that child.

In the first phase of selection, 12 people meeting the criteria for participation were identified, and individual contacts were made to collect further personal data and assess the availability of the volunteers to participate in the focus-group.

Finally, a group of 5 women was constituted and formal written consent was obtained from all participants. These women were aged between 31 and 44 years old; they were all highly educated (degree or more) and professionally active at the moment of the focus group. They came from different regions of the Portuguese territory, and they were all living with their partners (the fathers of their children). See Table 1.

**Table 1:** Characterization of participants.

**Name**	**Age**	**Academic degree**	**Child's age**	**Breastfeeding Status**	**Place of residence**
Alice	31	Master	19 months	Still breastfeeding	Northern region. Up to a medium sized city.
Arleen	37	Bachelor	2 years and 10 months	Still breastfeeding	Center region. Up to a medium sized city.
Linda	40	Master	1 year and 10 months	Still breastfeeding	Southern region. Small village.
Caroline	44	Bachelor	19 months	Breastfeeding up to 3.5 months	Lisbon metropolitan area
Lisa	44	Bachelor	3 years	Breastfeeding up to 22 months	Southern region. Up to medium sized village.

### 2.3. Analysis

The focus group was video-recorded and then transcribed. The transcription was complemented, when relevant, by descriptions from the authors. Transcripts of the recordings were coded in order to synthesize and explain larger segments of data, linking them to the central dimensions of discourse.

To analyze data, we performed a combination of inductive and deductive content analysis (Moretti et al., 2011). Sometimes, the coding process found a direct correspondence with theoretically derived dimensions that were previously defined in the script of the interview (deductive approach); but, in other occasions, new dimensions or categories emerged directly from the data instead (inductive approach), thereby launching new clues to the study of breastfeeding practices. These dimensions allowed us to organize the collected empirical material, establishing the basis for its analysis and interpretation (Silverman, 2000).

In the case of a qualitative study involving a limited number of participants and also a single moment of empirical inquiry, results must be acknowledged as of a purely exploratory nature and are intended to provide a starting point to further exploration in the future.

## 3. Results and discussion

### 3.1. Constructing the decision to breastfeed: Why to start and when to stop?

Previous family experiences seem to play an important role in the decision to breastfeed, and even when it is not an issue in the family, the experience with a significant other, namely a close friend, weighs on the decision to breastfeed. Alice mentioned that her grandmother, her mother, her sister, all breastfed. Even her sister's difficult experience was a source of motivation for her, as it led her to understand that “breastfeeding is possible, but it can be challenging.” That led her to keep as informed as possible about the process.

Arleen also mentioned the family experience, given that she and her sisters were breastfed, the youngest one until she was 5 years old. Lisa did not have this reference in her family. She confessed that, at the age of 41, she knew very little about pregnancy and motherhood. However, the fact that she had shared a house with a couple of friends who were parents, allowed her to follow the baby's first 2 years (and the difficulties related to childcare) and gave her the impression of “having attended a mini-course before getting pregnant.”

The decision to breastfeed was, for all of them, understood as the best option, the most natural, the one they had desired since pregnancy. None of them questioned the possibility of breastfeeding. Deciding to do so did not involve a great deal of reflexivity. Similarly to the participants in the research conducted by Andrews and Knaak (2013), breastfeeding was a taken for granted decision.

“When I knew I was pregnant, in my mind there was no chance of not breastfeeding” (Caroline).

“Breastfeeding was never a question. (...) It was always seen as normal, it was a non-issue” (Arleen).

“For me it was very obvious [to choose to breastfeed]” (Linda).

“In my family it was very normal, there was no question that it was not possible to breastfeed” (Alice).

The accounts produced around the “choice” alluded to the naturalness of breastfeeding and its benefits for the baby's health and wellbeing, advocating the idea that what is natural is good. Only one of them mentioned breastfeeding as reinforcing the affective bond that unites her to the baby. The decision-making to breastfeed involved different levels of expectations, with some women putting a lot of pressure on themselves and on their performance, while others simply believed in a process that they considered natural and normal. For some women, the search for information was a key factor to feel confident about their performance.

“It is part of the natural law of life, and I naturally thought about it. (...) I really wanted to breastfeed, I wanted to breastfeed him at least until he was 1 year old. I thought I would do as much as possible, but if it wasn't possible, then so be it” (Caroline).

“Regarding breastfeeding, I read a lot, I talked to friends, I informed myself a lot. I wanted the process to be as natural as possible (...). Between giving milk that is not natural and the possibility of giving my own, I was scared to give something that was not natural. (...) I did not have many expectations, because I felt confident with this thing of reading, informing myself, talking. So, I thought it was going to go well. If I didn't make it, I was going to be very sad” (Linda).

“I had confidence postpartum because I thought it was the best thing I could do for my child. My milk was the best food I could give my child. The information I sought was to shape me as the best mother for my child. If I failed, if I made mistakes, I was going to fail as a mother” (Alice).

“I thought 6 months was the minimum [to breastfeed], but with COVID-19 I kept putting it off because I thought that would be important for his immunity. The truth is that he is 3 years old and has never been sick. And I am glad to have contributed to that” (Lisa).

It is particularly noticeable in these statements: (i) the definition of breast milk as nutritionally superior (the “best food,” as opposed to “a thing” that is not natural) and beneficial for the baby's health; (ii) a clear focus on milk as a product; (iii) the definition of breastfeeding as a natural act; (iv) the prescription of a desirable period for breastfeeding. All these aspects are clearly associated with the process of medicalization of breastfeeding (Andrews and Knaak, 2013; Torres, 2014a), entailing the internalization of a biomedically oriented pro-breastfeeding discourse, which is mobilized by the participants to account for their decision. As Faircloth (2017, p. 22) states “mothers are accountable for the choice they make both within and between feeding alternatives (breast milk or formula).” In fact, mothers are corresponding to what Schmidt et al. (2022) consider to be neoliberal demands of self responsibility and self-optimization, framing mothering as a highly individualized performance and mothers as accountable subjects responsible for their choices and for the outcomes of those choices.

The search for information was a common aspect to all participants' pathways, albeit with different levels of involvement and diversification of sources. Alice is the one that denotes the most active and committed search for information, and reproduces the technical language usually used in informative sources about the benefits of breastfeeding. As mentioned above, this participant didn't want to repeat her sister's breastfeeding story, so she read everything she could, attended courses on Baby Led Weaning, and resorted to a lactation consultant. In fact, it was surprising to note that all participants, at some point, had contact or, at least, recognized the figure of the certified lactation consultants.

Withholding information seems to be a way of introducing some control in a process which, they acknowledge, is full of uncertainties and new to all of them. Schmidt et al. (2022) state that in their attempts to be in control, mothers often follow information they collected from experts and from other mothers. They feel that success relies a lot on themselves, and that they should do everything in their power to make it go as smoothly as possible, which in a way reveals the socially shared notion that breastfeeding is, above all, a matter of personal choice and commitment.

“I read a lot, talked to friends, got a lot of information. I wanted the process to be as natural as possible, so I needed to understand the process, the mechanism and all that stuff. Breastfeeding was a non-issue. I didn't have high expectations, because I felt confident reading, informing myself and speaking about it. I knew it would go well” (Linda).

“I felt similar to Linda, reading a lot, informing myself a lot. (...) I knew [breastfeeding] was normal and who should I look for in case it didn't work. I have a tendency to control things and I knew that this mostly depended on me and that I would find a way to work” (Arleen).

The participants' accounts illustrate a certain ambivalence that is also present in the medical discourse (Carter, 1995; Andrews and Knaak, 2013), since although they consider breastfeeding to be something natural, they feel the need to seek scientific information and to resort to the intervention of experts to support them.

This recognition of breastfeeding as natural and, therefore, desirable and the best option for the baby's health and future development, is very much associated with the construct of good motherhood (Schmied and Lupton, 2001; Badinter, 2010; Faircloth, 2017; Schmidt et al., 2022). And, therefore, some authors associate it with a moral burden, which generates feelings of guilt and shame (Carter, 1995; Blum, 1999; Taylor and Wallace, 2012; Thomson et al., 2015; Jackson et al., 2021; Doonan, 2022; Naylor, 2022). Schmidt et al. (2022) point out that one of the emotional responses most described in the studies included in their systematic review is the feeling of guilt. For them, guilt acts as a regulating force in mother's lives as they strive to adhere to the norm of being responsible for the development, health and well-being of their children. Some of our participants revealed that they felt anxious about their future breastfeeding performance, and one of them even considered that if she failed, if she made a mistake in breastfeeding, she would be failing as a mother.

Differently to the decision to breastfeed, the decision to stop breastfeeding seems to be subject to much more reflection, as it is viewed with some reluctance and as a source of anxiety and suffering. Of all the participants, only two had stopped breastfeeding at the moment of the interview, one of them not by choice. In fact, apart from Caroline, who only breastfed until her son was 3.5 months old, they all breastfed (or are breastfeeding) beyond the first year of the children. Only Lisa decided to stop breastfeed her son, when he was about 2 years old. The remaining children are still breastfed: Alice's son is 19 months old; Arleen' son is 2 years and 11 months old; Linda's daughter is 23 months old.

In Caroline's case, the decision to stop breastfeeding resulted from an experience that she recognized as hard for her and the baby. In her own words, she “tried everything”: she spoke with the pediatrician, with friends, and she tried her best to overcome difficulties.

“I tried for a month, but he cried, it was difficult for both of us. At some point, the situation was bad for the baby and I chose to stop. When I decided to stop, I obviously was sad, feeling a little down. But I decided not to make a drama, nor to become like some mothers I see around me, with feelings of guilt” (Caroline).

As a matter of fact, all participants revealed a high level of commitment, all of them expressed being strongly motivated to do everything within their reach to have the best possible performance, the one that came closest to the norm. This commitment can be partially explained by the socio-economic status of these women (Schmied and Lupton, 2001), all of them with high education qualifications and belonging to the middle class.

Lisa decided to stop because she felt it was time to think of herself as a person and as a woman again, as if breastfeeding suspended other dimensions of oneself and one's life which, at a certain point, depending on each woman, is necessary to recover: “I was feeling very tired. I became a mother late, and this is a very demanding process. I was a bit exhausted, wanting to take back my body and to restore some energy to myself. I also felt that this whole breastfeeding process lowered my libido a lot, and I thought it was time to recover other pleasures, in addition to the pleasure of motherhood” (Lisa).

Lisa's option seems to suggest a desire to recover some autonomy, to regain control over her body, to allocate energy to herself, to restore her sexuality. Contrary to the process described by Schmied and Lupton (2001, p. 240), it appears to be a repositioning of the frontiers between self and Other (the baby). She believes that from the moment the mother decides to stop, “there is a kind of understanding, a kind of language of the unspoken between the mother and the child. But one has to believe in that. Because I took a long time, and so, I kept postponing it” (Lisa).

Two of the participants said they had already recognized some signs of weaning, but not immediately. Arleen referred to the feeling of tiredness, a huge drop in energy, which happened when her son was around 2.5 years old. However, she emphasized that the decision to stop is hers, that it is up to her to decide, even admitting to be facing external pressures to stop breastfeeding from people that are close to her, namely her husband (which hurts her the most). Of all the times she thought of weaning, she realizes now, it was mostly due to external pressure.

It is interesting to notice that more than social pressure to breastfeed, which was only felt by one of the participants, the most mentioned type of pressure and the one that had the most impact on mothers, was the pressure to weaning, to stop breastfeeding. Indeed, the same society that pressures women to breastfeed, stressing the need to fulfill a biological and social purpose, underlying the breastfeeding benefits for mother and baby tends, under some circumstances, to pressure in the opposite direction. There seems to be a time frame, a socially accepted limit for breastfeeding, after which it is regarded as unnatural, not normal, not desirable and cause of embarrassment. Actually, it comes to be understood in a way that is completely opposed to the social valuation and representation of breastfeeding and a sign of “bad mothering.”

“I still feel that extended breastfeeding makes people uncomfortable. I hear this a lot, especially from my employer: ‘Oh, you still breastfeed. That's becoming an addiction. At night, she should sleep peacefully in her little room, that's not good. Like this, nobody rests’. I make my best smile, and I don't care! (...) For my parents, it is especially difficult to understand what free demand is. I live this thing of “she's breastfeeding again, she's always breastfeeding”” (Linda).

Arleen told us about an episode in which, after holding her son in the nursery and breastfeeding him, the nursery teacher asked her if she would prefer to go to a more private room. She told her that she was fine, but the teacher insisted that it was better, so as not to disturb the other parents. In this case, the discomfort caused by breastfeeding does not stem from the child's age, but from the idea that breastfeeding should be done in private, with modesty, because it is something that breaks the rules of social interaction, largely due to the sexual connotation of the woman's breasts. In every paper of their systematic literature review, Grant et al. (2022) identified discourses suggesting that breastfeeding was viewed as an antisocial act to be conducted in private only. Such discourses were largely related to the sexualization of breasts. Naylor (2022) states that the topic of breastfeeding in public is not new, and there is a well- established literature about public shaming and breastfeeding.

The pressure to stop is particularly felt by these women when it comes from the people closest to them, who make a point of demonstrating that it is necessary to put an end to this practice. Arleen, who coped well with the teacher's call to attention and even wrote a letter to the headmaster asking for an explanation, has much more difficulty in dealing with the pressure from her husband. Also Dowling and Brown (2012) identified pressures coming from women's partners to weaning, especially in cases of extended breastfeeding.

“I often hear ‘But is he still breastfeeding? He's too old to still breastfeed.’ And I'm not talking about outsiders. The opinions of those closest to me are the most difficult ones, and even when my husband says that this has to stop, because he doesn't sleep. In those moments I really feel a stab in the back. (...) I feel this pressure from my husband, but the times I wake up, he's sleeping. And even if the nights are bad, I have the right to continue not sleeping and breastfeeding. But it's getting harder and harder because I feel like I have no way to argue. How do I say that this thing I want to do is something I want to do?” (Arleen).

Extended (prolonged/long term) and on-demand breastfeeding is a demanding process for mothers. All mentioned the fatigue, the sleepless nights. However, all currently breastfeeding mothers also said that they still do not feel that it is time to stop, although they have already thought about it, although they are feeling exhausted. In addition to this demanding process, women also have to manage external pressures to weaning, and constantly justify and defend their will to breastfeed beyond what is socially considered the adequate and acceptable time. In Lisa's case, the close pressure to stop came from her father, and she refused the implicit judgment that his opinion implied.

“In relation to the end of breastfeeding, around the year, I began to feel various pressures. The one that stuck with me the most was the one from my father. It seemed like a question of masculinity, that I was uneducating my son, keeping him under his mother's skirts. I felt it as a criticism of my relationship with the child. And I drew a boundary, 'this is not your business!' That offended me a lot at the time” (Lisa).

Lee (2018) considers that breastfeeding of older children can cause discomfort. She draws on the contribution of some authors, who discuss this issue and state that mothers who breastfeed for longer periods are frequently accused of being indulgent and putting their personal pleasure and emotional needs ahead of child's welfare.

### 3.2. Expertises and sources of information

Paradoxically to the idea that breastfeeding is a natural attribute and practice, there is an increasing recognition of the need for effective professional support when a woman decides to breastfeed. In contemporary western societies, understanding such a paradox is only possible by taking into account broader phenomena such as the medicalization of motherhood, risk management, and the processes of legitimization of knowledge.

As mentioned above, the search and access to information are important aspects of women's decisions around breastfeeding. Reflecting a broader trend in society, the need for information and knowledge is a constant in women's sharings and it is something that all of them attribute to their way of being and behavior as mothers.

“I feel a lot of need for information regarding how things work. [I need to know] what is in my power to do everything right!” (Arleen).

Participants mentioned to have benefited from different types of support and sources of information along their breastfeeding trajectories. Friends, family and communities of support were some of the sources mentioned by the group. But what was actually quite prevailing in their accounts was the role of the experts, and particularly the lactation consultants. Breastfeeding is strongly connected with the expectation of making an informed choice based on scientific knowledge, information provided by official campaigns (Schmidt et al., 2022) and lactation experts.

Actually, all the women, at the moment of the interview, declared to know the figure of the lactation consultant, some having had a direct contact with these specialists in a given moment of their breastfeeding pathways. In these cases, speeches tended to highly value the consultant's support, considering it as a crucial factor for the success of the breastfeeding experience.

“In my emergency contacts, I had this lactation consultant that I could contact in case of need. The fact of knowing that it's not normal to hurt, that different positions can be tried, etc., etc. was reassuring” (Arleen).

“I got a lot of information and it gave me confidence (…). The contact I had with a lactation consultant was crucial” (Alice).

Lactation consultants are a new (and not fully established) occupation, built upon traditional forms of care work. In a society adverse to risk (Lash, 2002), parents and particularly mothers turn to specialists for technically supported solutions that minimize their insecurities and doubts, and ensure, in a non-individualized way, the responsibility of caring for the child. These specialists, mostly women, build their legitimacy on taking care of both the mother and the baby, acting in a highly feminized context. Their function is actually related to attributes socially considered “innate to women,” such as nurturing, comforting, encouraging or caring (Torres, 2014b).

The social legitimacy of these professionals must be understood in a cultural scenario where traditional support tends to be replaced by paid expert systems in a logic of outsourcing and expertise of care. And, in fact, some of the participants refer to the lack of traditional support for their mothering practices—family, friends, close community, neighborhood—as an explanation to seek specialized support, as the one that is provided by lactation consultants.

“I found it very strange not to share this moment in life. What an enormous silence there is among women! I don't have siblings; I don't have close cousins either. In my family I did not live other experiences of motherhood, in my friendships neither (...). I was a little disappointed [with the lack of a close support network]” (Lisa).

### 3.3. Conciliating breastfeeding with other dimensions of life: Work-life and couple intimacy

Conciliation between motherhood and other spheres of life was another topic discussed by the group. In particular, the discussion focused mainly on the balance between maternal practices and two other women's life dimensions: work-life and intimacy.

#### 3.3.1. Work-life

Despite scientific recommendations for exclusive breastfeeding until 6 months of age and complementary breastfeeding to 2 years of age, breastfeeding abandonment rates increase with time, and one of the main reasons is that women go back to work (Chordá, 2020).

Among the group, experiences of conciliation between the return to work and breastfeeding were diverse, but they all have required ponderation, changes and adaptation, which confirms the relevance of this subject to the approach of breastfeed practices.

All employed women who were breastfeeding when returning to work benefited (some continue to benefit) from the right, legally granted in Portugal to lactating working mothers, to reduce working hours. Women working in smaller contexts, with greater proximity to their peers and employers were the ones who, not surprisingly, felt greater resistance from their professional context to the exercise of their rights and to perform the conciliation between professional and maternal duties. Alice, who was committed to keeping her 5 months baby breastfeeding on free demand when she returned to work, described the demanding logistics this choice represented:

“Before returning to work, I thought “I will pump milk each day, and, the day after, my parents will give it to my baby, and everything's gonna be fine!” But, of course, when a person goes back to work, there is so much change that it is really challenging. [For a moment] I even thought I was going to give up and that I wouldn't have enough milk for my boy! But, well, we continued and, later on, I was able to store some milk. I had a stock that was not too big, but it was enough to stay more comfortable” (Alice).

Beside requiring family support, the logistics of storing the milk on a daily basis also interfered with the professional context and forced a reorganization of working hours, with direct impact on break times and on professional relationships. In fact, as Faircloth (2017, p. 38) suggests, “in the social and economic context of post-industrialized societies, following perceived natural patterns of lactation creates a “cultural contradiction” for the woman doing it.”

“I felt pressure from my employer because I pumped milk during my breaks, then I came home to lunch and breastfed my baby. Then, in the afternoon, I pumped again during the break. (...) It seemed like I was always expressing milk [in the workplace], but I only did it during my breaks and sometimes I didn't even stop working. But I received some comments that, in my opinion, were a little unnecessary. (..) When my son was already 16 months old, I realized that I was under too much pressure at work and (...), since I had some milk in stock, I decided to stop pumping milk at work” (Alice).

Schmidt et al. (2022) describe five contemporary norms of motherhood that are related to five types of mothers. One of them is the working mother, connected to the norm of integrating employment into mothering. These mothers are expected to align their professional activities with their mothering by responsibly adjusting the two life spheres, which can make them feel pressured to conform to expectations that can be contradictory and conflicting. Even if Alice's right to enjoy breastfeed breaks was not openly contested, she felt that it was not a welcome practice either, which led her to stop trying to integrate breast milk production and expression with employment commitments. This effort to integrate these two aspects is recognized by Johnson (2019, p. 424) as a form of “maternal body work.”

On the other hand, women working in big companies seem, in a certain way, to have fewer problems in seeing their rights fulfilled. As Arleen explained, in bigger companies, all the aspects related to parental leaves are handled administratively, with no room for any social judgments or questions of morality.

“My company is a very large company and in this case I think it helps. The HR department deals with the issue of licenses, and they don't even know me. I send the email with the medical certification that I'm still breastfeeding, and no one asks questions, no one asks for explanations, nothing! Even when I was pregnant, they had prepared a small room for the mothers to pump milk, with a fridge and all the conditions. I never got to use them, because shortly after having the baby the pandemic began, but in that sense, it has been great! I don't have the slightest thing to point out!” (Arleen).

Finally, for self-employed mothers and for women who work in more flexible work arrangements, the reconciliation between returning to work and breastfeeding is quite particular, since this context seems, in part, to avoid or mitigate the effects of the anxiety that is common to breastfeeding women returning to work.

“I started working at age 23 and have never stopped working. When I got pregnant, I organized my life to stop working for the entire first year of my baby. It may seem counterintuitive, but I decided to take advantage of this time to slow down, to accept that life was going to be different” (Lisa).

During that first year, Lisa still cooperated occasionally in some projects, but always by choice. She confessed she can't imagine what it would have been like to go back to work intensively with her son so young. And it is, precisely, this feeling that makes her admire women who, under a huge effort, manage to do so.

“That would have been very bad. Very bad! (...) I feel that I would not have known how to manage breastfeeding under these circumstances, and I have a lot of admiration for women who manage to do it” (Lisa).

With greater or lesser pragmatic difficulties in conciliating, all participants agreed on the idea that their relationship with work has been transformed with the experience of motherhood. Accounts, in some cases, came to recognize changes and constraints in one's productivity and the difficulty in recovering the work performance of other times, but in general no one considered that, in the end, has become a worst worker.

“First, I went through a phase feeling very incompetent [laughs], but then I think I started to be able to manage things better” (Lisa).

“The same capacity for work [I don't have], at all! The same commitment, yes. (...) I don't feel I've become any less competent or that I do my job worse. Maybe I don't spend as much time working as I used to, but I also don't spend less than I should. (...) I'm still a workaholic, I'm just in more of a hurry to get home now!” (Caroline).

There are even those, like Arleen, who recognized that motherhood has brought a healthier work-life balance, putting things in perspective, relativizing problems and rearranging priorities.

“Motherhood, despite making everything heavier, made me lighter [regarding work], curiously. I used to get very hung up on details and now, if there's a problem, ok it's annoying, but it's not the end of the world. I didn't stop working my hours, of course, and I do it with all my effort, but I do it more lightly than I did before. [In my work] there are many things that I can't control and that used to weigh me down. And now those things don't weigh me the same way” (Arleen).

#### 3.3.2. Intimacy and couple life

Concerning the impacts of mothering practices (particularly breastfeeding) on couples' life and romantic relationships, accounts were variable, but, once again, the idea that becoming a mother is a total experience which impacts on all levels of existence (including intimacy) seemed to be consensual within the group.

In most cases, especially among the youngest participants, accounts emphasized the difficulties of the relationship felt in the early days of motherhood. Distance from their partners, feelings of incomprehension and frustration were aspects shared by the participants.

“Having a child is like having a truck run over romantic relationships. It brings a lot of tension or, rather, it puts a spotlight on them... it's impossible not to see those tensions because they are quite evident. I felt a great distance between me and my husband in the first 6 months” (Arleen).

The idea of the mother remaining in a bubble with the child in the postpartum, in an arrangement that excludes the father, was described by some of the women. In this context, breastfeeding seems to be acknowledged as the cement that glues the mother-baby dyad, invariably relegating the father to a secondary role. Breastfeeding can shift the dyad of male-female to female-child (Bartlett, 2005). Women try to navigate lactation as mothers and lovers (Carathers, 2017). To overcome these difficulties, communication in the couple is considered essential, but sometimes not enough, as described by a participant:

“Because I was completely focused on the baby, we really had to do couple therapy for a while. [The therapy] helped a lot. (...) Then, as we also mature, as people and as a couple, we also manage to work these things in a slightly different way, and I think we are in a better place now!” (Arleen).

This distancing is particularly felt in the couple's sexual life. For these women, at some point, the experience of childbirth and other embodied maternal practices, such as breastfeeding, colonized their bodies and dispositions, suspending the romantic and sexual bond that linked them to their partners. Also Carathers (2017), in her research, says that mothers described a process of maternal disembodiment, segmenting their identities in relation to competing demands made on their body by their babies and their partners. Most of them expressed ambiguity regarding the management of their breasts as both sexual objects and feeding instruments. As Schmied and Lupton (2001, p. 245) stated, “motherhood is associated with one type of love and sexuality with another,” and for some women, apparently, their coexistence is hard to handle.

“In the beginning, when my husband made [intimate] advances, I couldn't turn off the chip. I was very programmed to take care of the baby (...) It was a bit difficult, but with communication (because I was open with my husband and told him how it was working or rather, how it was NOT working) and his comprehension things then flowed” (Alice).

With regard to breastfeeding, in particular, participants referred to changes in the way they acknowledged and experienced their breasts in intimacy. Breast milk, being the baby's source of food, encloses the female breast in a domain that is accessible only to the baby, and opposed to any erotic meaning, for these women. According to literature (e.g., Davis, 1997; Sandre-Pereira, 2003; Pissolato et al., 2016), the changes in a woman's body when becoming a mother and her special bond with the baby during the breastfeeding period can be experienced by the couple differently. The perceived impossibility of coexistence in the same body of the “mother” and the “woman” can generate a conflicting situation for women and for couples. Managing breastfeeding and sexuality illustrates yet another contradiction experienced by women as they try to reconcile the demands of hypersexualized body images and expectations with realities of motherhood and lactating breasts (Carathers, 2017).

These conflicting and deeply embodied tensions emerged, with particular strength, from the discourses of two participants:

“Like Alice, I also feel that the breast, which used to be, so to speak, an object of pleasure, has become completely dominated by the child. (...) For me it was very difficult, because the breast for me has always been a very erogenous zone and after childbirth that disappeared. It cost me a lot because for me it was always a part of my body that gave me a lot of pleasure. It was as if breastfeeding had stolen that from me” (Arleen).

However, it is curious to note that, even facing difficulties, women do not fail to look for opportunities to improve their relationships. In an attempt to conciliate maternal practices with the wellbeing of romantic relationships, recovering pleasure and the sovereignty over their bodies, women work hard on relationships, communicating, keeping an active watch over the reality that surrounds them. It is also the willingness to look at the positive side of situations and to not blame motherhood for constraints that their testimonies express:

“Although I didn't have a vaginal birth (…), I felt a huge transformation in my sexuality and I think this has to do with my transformation as a person. This is how I am, I know what I want and what I don't want. And maybe this is also what you get from being a mother, isn't it? (...) Perhaps, sexual intercourse is not as spontaneous as it used to be, because of our son, but that doesn't make it any less good and, well, it's fun to discover that! (...) [Also,] the fact that my breasts were stolen for another purpose also opened the door to [intimately] explore other things we had not explored before, and it was very interesting. This discovery has been very interesting!” (Arleen).

When it comes to discuss the balance between different spheres of life, despite the difficulties inherent in conciliation (which all participants without exception reported), it is interesting to see how the discourses tended to be eminently positive and optimist, as if motherhood came to reestablish an order of priorities that was and continues to be beneficial to these mothers. Lisa's words were very enlightening about this.

“I feel like I've become a much more focused person [with motherhood]. Much more focused! Now there are things that don't interest me at all. Things that before motherhood were around and distracted me and, therefore, motherhood, despite having less time, brought me a certain effectiveness, a certain clarity, which I don't know how long it will last, but that I am enjoying a lot, so far [laughs]” (Lisa).

Also interesting to see is how mothers age or their stage in the lifespan may be considered a factor in their ability to abdicate, their selflessness and, ultimately, their willingness to accept the limits. Concretely, Lisa, who was 41 years old when her son was born, considered positively the fact of becoming a mother lately, in a moment of life when it is assumed that people already feel fulfilled by the richness of their life experiences. In this sense, motherhood does not compete with other ambitions, because, somehow, these would already be satisfied. In her words:

“I'm glad I didn't have children before. I think, considering my personality, it would have been difficult for me to conciliate. But, right now, I never get upset if I can't do something. It's okay, maybe because I feel like I've done a lot of things throughout my life. Conciliation is easier, also in a couple. (...) [When self-fulfilled] everyone has a greater capacity to give up” (Lisa).

### 3.4. The role of the father in breastfeeding

Literature on the factors that contribute to successful breastfeeding often highlights the importance of high-quality professional guidance as an element of breastfeeding success, but less research has explored the role of fathers in both women's decision to initiate and their ability to continue breastfeeding (Brown and Davies, 2014). Nevertheless, there's already evidence that fathers' attitudes and actions can positively or negatively impact on mothers' intentions to breastfeed, breastfeeding duration and exclusivity (McCarter-Spaulding, 2008; Abu-Abbas et al., 2016; deMontigny et al., 2018; Hansen et al., 2018; Hounsome and Dowling, 2018; Crippa et al., 2021; Johnson and Slauson-Blevins, 2022). Most of this literature explores the father's perspective, experiences and attitudes regarding breastfeeding. Similarly to Johnson and Slauson-Blevins (2022), we address women's perceptions of partner's support, stressing (as they do) the importance of the receiver's interpretation of support.

Three main forms of social support provided by male partners have been identified in the literature: emotional, informational, and instrumental (Johnson and Slauson-Blevins, 2022). In general, women agreed that their partners collaborated and supported their experiences and decisions regarding breastfeeding. However, there are differences that distinguish the type of support and the effective role of their partners in the process. In most cases, this support was described as motivational (emotional—providing love, encouragement, empathy) but not really a practical support (instrumental—providing help with domestic tasks), as illustrated by the following quote:

“He didn't help me with the latch or this kind of thing, but he was my right hand. When I woke up at night, he woke up too. He always followed me. (...) He was always there by my side and supporting me, so that things went well” (Caroline).

In the case of Alice, the partner's support was described as almost specialized (informational—providing information and “expert” help) in the sense of an effective and practical support, very involved in the dynamics and practices of breastfeeding:

“I often say that in the first few weeks he was my lactation consultant! He was the one carrying the bags of hot seeds when the milk came up, he was crucial to successful breastfeeding... He was outstanding!” (Alice).

On the other hand, support may refer to the father's availability, in the absence of the mother, to ensure the continuity of breastfeeding, in an attitude of great encouragement to the fulfillment of the mother's personal and professional projects, clearly assuming an instrumental support, replacing (even if only temporarily) the mother in domestic and caregiving tasks That was the case of Lisa:

“I always felt an enormous support [from my partner]. When our baby was 4 months old, I had a proposal to present my work abroad (…) and he encouraged me a lot to go, as this implied a huge effort for him, which was to spend 3 days with the baby, without the breast. And that also meant that I had to pump a lot of milk [in advance] and I felt very tired. (…). I felt that he encouraged me a lot” (Lisa).

Even though their descriptions were mainly related to the early stages of the breastfeeding experience, one of the participants shared a certain degradation of her partner's support over time, mentioning feeling pressured by her partner, in a more advanced stage of their breastfeeding trajectory, to anticipate the weaning of her child. Johnson and Slauson-Blevins (2022) findings point to the possibility that father's support may decrease over time with the age of the child, eventually giving way to a conditional support. Despite being disappointed with her partner's attitude, she does not fail to assume what she considers to be her own responsibility for the unequal couple division of the breastfeeding duties and the childcare.

“I felt that in the beginning there was much more participation and support [from my husband]. (...) Then, with exhaustion and the passage of time, he stopped waking up at night or no longer got up every time I did. (...) It's difficult because when I put the baby to the breast, he falls asleep, but if the father has to cradle him, it takes forever to fall asleep, so I also had responsibility because I didn't promote his participation. It is something we will certainly do differently next time!” (Arleen).

Women's perceptions regarding the role and support of their partners in breastfeeding give an idea of the diversity of male attitudes and practices in a context of changing expectations about fatherhood. The analysis comes to reveal how the practices and attitudes of the partners are diverse, not only because, like women, men are different from each other, but also because contexts and relationships change over time, shaping attitudes and, ultimately, the conditions under which breastfeeding experiences take place.

### 3.5. Changes in self-concept and processes of identity work

Some literature suggests that women change their identities in various ways when they become mothers. According to Laney et al. (2015), in the transition to motherhood, women face a complex process that may involve a period of self-loss and identity reconfiguration. More than in any other type of relationship, in motherhood, women often expand their identities to include another person within the limits of the self and consciousness.

Although, in general, the transition to motherhood is associated with joy, becoming a mother and mothering, in addition to undergoing important physical and emotional transformations, requires adapting to new roles, which can generate feelings of anxiety, conflict, and ambivalence (Brown, 2010).

Among the group, motherhood and breastfeeding are generally described as positive and empowering experiences. According to O'Reilly (2004, 2019), the disadvantageous conditions of motherhood and the oppression that characterizes motherhood as a patriarchal institution should not overshadow the potentiality of empowered mothering, nor the possibility for women to live motherhood from a position of agency, authority, and autonomy. In the case of participants, the idea of empowerment appears strongly associated with important changes, brought about by motherhood, in terms of women's personal and social identities. Concerning these changes, all the women agreed on the idea that motherhood (at this point, they do not refer only to breastfeeding) gave them the ability to put problems into perspective and face reality and difficulties in a less dramatic and defeatist perspective.

“There are a lot of things that change in life with motherhood, especially, the way we see problems. I now relativize the problems much more. (...) And I have the feeling that this is over, that there is no going back (...). Maybe, before I gave much more importance to certain problems and now I [relativize them]. (...) [Before becoming a mother] I was more dramatic, I think” (Caroline).

Along with this ability to relativize situations, a participant (Lisa) also mentioned the idea that the transition to motherhood represented a real acquisition of new skills for life.

“Due to my work, I often have to speak in public and this relationship with the public has always been a painful thing. But after this experience, the first year [as a mother], honestly or extraordinarily, there are things that are no longer difficult for me. I have the feeling that this makes me look at the idea of female power, because it really goes beyond relativizing. (…) It's like looking at the world through another lens” (Lisa).

Breastfeeding was also described as an embodied practice. In the discussion there were many accounts about the idea of self-image and the relationship with the body. Some women shared the need, felt at a certain moment, to recover the sovereignty over their own bodies. Changes and impacts caused by pregnancy and breastfeeding on bodies were enthusiastically described. At this point, speeches varied from the simple acceptance of the body changes to the praise for the physical transformations these women have undergone.

“I loved being pregnant, I felt beautiful, I felt sexy, I had amazing hair, amazing tits. I felt like a goddess, and it was a really good experience. Then I experienced a birth that I didn't want at all, and it was not easy to deal with. I've been trying to process all these things for almost 3 years now. I feel myself refocusing, accepting what I am now, but I know that I am not the same as I was and even my body is very different. I'm also older, right? But I'm strangely comfortable with it. I'm not trying to be anything else, I'm just comfortable with who I am” (Arleen).

“I have always had a complex relationship with my breasts. [Breastfeeding and mothering] helped me to overcome some things in my relationship with my body that, despite my age, were still there. This whole process, which is extremely physical, contributed to the acceptance, to understand how the body itself can be challenged. And after 3 years, despite this speech, I feel that I am only now getting my body back” (Lisa).

From the participant's testimonies emerged the idea of breastfeeding as a trigger for the identification/recognition of common traits among women and sorority. However, apart from Alice, no other participant declared having changed her way of acknowledging, empathizing or relating to other women, due to the experiences of breastfeeding or mothering.

“When we give birth, another woman is reborn and that's what I felt. I felt very empowered and confident, no matter what. (...) I recognize myself in the idea of seeing things lighter and having the ability to put myself in the shoes of other women. Before my son was born, I had that fear of failing as a mother, and today I know that doesn't exist. There are no mistakes, no failures in motherhood. It is what it is! We all do our best and I can see that now, in motherhood” (Alice).

“I don't think the way I look at other women has changed because they are mothers or not. I don't notice that becoming a mother has impacted my relationships [with other women]” (Caroline).

Even so, a participant (Arleen) referred to have created new social and friendship networks due to the common experience of breastfeeding.

“The experience of breastfeeding allowed me to get closer to people that, otherwise, I would not have met. I don't want to use the cliché 'I found my tribe' and things like that because it has nothing to do with me, but it has been interesting to meet people, despite being very different from me, with whom I share common interests” (Arleen).

This reference to a “breastfeeding community,” when discussing the impact of motherhood and breastfeeding on identity changes, is quite significant for our analysis. First, because it draws attention to issues like the need of belonging and the identification with the other. Then, because this testimony highlights, once again, the positive changes and opportunities brought about by motherhood to these women in terms of self-concept and daily lives. It seems that, despite the difficulties, participants are committed to represent motherhood and, in this case, breastfeeding as rewarding potentials for change.

## 4. Concluding remarks

Starting from a framework that sought to identify some of the main theoretical debates around breastfeeding and being based on results from a focus group with five highly educated working mothers of a first child, this paper aimed to identity features of prevailing breastfeeding regimes in Portuguese society, while considering the personal circumstances and dilemmas experienced in the first person. Although exploratory and based on limited empirical data, our analysis allowed us to capture the diversity of breastfeeding experiences carried by the circumstances of these women and to reinforce the assumption that breastfeeding cannot be analyzed and understood separately from the different dimensions of women's lives.

In particular, five dimensions were identified, around which the analysis was built: constructing the decision to breastfeed; expertises and sources of information; conciliation; the role of the father; changes in self-concept and identities. To conclude, we can now summarize the main findings that resulted from our analysis, throughout this paper.

For these women, the decision to breastfeed did not result from great reflexivity, being understood as the most natural and taken for granted option. Nevertheless, despite considering breastfeeding a natural attribute and practice, women validate and internalize the prevalent assumption that breastfeeding requires an effective professional support and the access to specialized information.

Women identify tensions in reconciling breastfeeding with other facets of life, particularly work-life and sexual intimacy. However, rather than placing the onus of such difficulties on motherhood, their discourses highlight the potential of motherhood in establishing a new order of priorities that is ultimately perceived as positive.

Concerning the role of their partners, participants recognize the importance of their support for the overall success of the breastfeeding experiences. The role of fathers can take different forms depending on the characteristics of individuals and relationships and it changes over time.

Finally, among women, breastfeeding, like motherhood in general, is considered an empowering experience with a strong impact on their lives and identities. The discourses point to an identity construction very much based on motherhood, sublimating the gains inherent to the changes. Once again, there is an almost ascetic understanding of the transition to motherhood, as an opportunity for the construction of a stronger self.

## Data availability statement

The raw data supporting the conclusions of this article will be made available by the authors, without undue reservation.

## Ethics statement

Ethical review and approval was not required for the study on human participants in accordance with the local legislation and institutional requirements. The participants provided their written informed consent to participate in this study.

## Author contributions

AA and DN have equally made a substantial, direct, and intellectual contribution to the work. VH contributed to the design of the study, the data collection, and part of the data analysis. All authors listed approved the work for publication.
